# Co-expression of Piwil2/Piwil4 in nucleus indicates poor prognosis of hepatocellular carcinoma

**DOI:** 10.18632/oncotarget.13491

**Published:** 2016-11-22

**Authors:** Guangping Zeng, Deying Zhang, Xing Liu, Qing Kang, Yiyao Fu, Bo Tang, Wenhao Guo, Yuanyuan Zhang, Guanghui Wei, Dawei He

**Affiliations:** ^1^ Department of Urology, Children's Hospital of Chongqing Medical University, Ministry of Education Key Laboratory of Child Development and Disorders, Key Laboratory of Pediatrics in Chongqing, Chongqing International Science and Technology Cooperation Center for Child Development and Disorders, Chongqing 400014, China; ^2^ Wake Forest Institute for Regenerative Medicine, Wake Forest University School of Medicine, Medical Center Blvd, Winston-Salem, North Carolina 27103, USA

**Keywords:** hepatocellular carcinoma, molecular chaperone, Piwil2, Piwil4, prognosis

## Abstract

**Purpose:**

This study aimed to explore the localization and expression of P-element-induced wimpy testis-like 2 (piwil2)/Piwil4 in hepatocellular carcinoma (HCC) tissues, and analyze the correlation between co-expression pattern and prognosis of HCC.

**Results:**

Piwil2 showed 100% positive expression in the cell nucleus, with the intensity higher than in the cytoplasm. Piwil4 showed a lower intensity of expression in the cell nucleus than in the cytoplasm. The molecular chaperone Piwil2/Piwil4 had four co-expression patterns: nuclear co-expression, nuclear and cytoplasmic co-expression, cytoplasmic co-expression, and non-coexpression. The survival rate and the overall survival sequentially increased. The prognostic phenotype of the nuclear co-expression of Piwil2/Piwil4 was worse than that of non-coexpression, and the intracellular localization and expression of Piwil2 and Piwil4 were not significantly different.

**Methods:**

HCC pathological tissue samples with follow-up information (90 cases) and 2 normal control liver tissues were collected and made into a 92-site microarray. The expression of Piwil2 and Piwil4 was detected using the immunofluorescence double staining method. The differences in the expression and location of Piwil2 and Piwil4 in tumor cells were explored, and the influence of such differences on the long-term survival rate of HCC was studied using Kaplan-Meier survival curve and log-rank test. The clinical staging was analyzed according to the HCC international TNM staging criteria.

**Conclusions:**

The nuclear co-expression of Piwil2/Piwil4 indicated that patients with HCC had a worse prognostic phenotype. The molecular chaperone Piwil2/Piwil4 seems promising as a molecular marker for prognosis judgment; a single marker (Piwil2/Piwil4) cannot be used for prognosis judgment.

## INTRODUCTION

Primary liver cancer is one of the most common malignant tumors of the digestive system, of which 80%-90% is hepatocellular carcinoma (HCC). The morbidity and fatality rates rank fifth and third, respectively, among the malignant tumors in the world [1, 2]. Radical excision is the main surgical treatment for liver cancer, but the high metastasis and relapse rates after operation severely affect the long-term survival of patients with HCC. P-element-induced wimpy testis (PIWI) protein plays an important role in stem cell self-renewal, spermatogenesis, RNA interference (RNAi), transposon silencing, transcriptional control, genetic recombination, genetic programming, and so on [3–5]. Piwil2 is the key regulation gene for self-renewal of germ stem cells, and it regulates the self-renewal of germ stem cells and orderly development of sperms cooperatively with molecular chaperone Piwil4 [6–8]. It has been reported that the expression of Piwil2 and Piwil4 in malignant tumors may be related to the occurrence, development, and prognosis of tumors [9–11]. Piwil2 protein activates the STAT3/Bcl-XL pathway to inhibit apoptosis, which is widely expressed in various tumors [12]. It may greatly promote the occurrence and metastasis of tumors [10, 13–15]. The expression level of Piwil4 protein is increased in soft tissue sarcoma and colon cancer, which is importantly related to poor prognosis and increased risk of metastasis [11, 16]. However, the influence of a single marker (Piwil2 or Piwil4) on the biological behavior and long-term survival, which is used to judge the development or prognosis of tumors, is very difficult. Furthermore, the reports on the correlation between the co-expression of Piwil2/Piwil4 and the occurrence, development, and prognosis of tumors are limited. As molecular chaperones, Piwil2/Piwil4 are co-expressed and localized in the tumor tissue. Whether they can be used as the indicator for tumor development or prognosis still needs further exploration. This study used a tissue chip (high efficiency and high throughput) [17] and immunofluorescent double staining to explore the localization and expression of Piwil2/Piwil4 in HCC tissues and analyze the correlation between co-expression pattern and prognosis.

## RESULTS

### Expression of Piwil2 and Piwil4 in normal liver tissue

**Figure 1:**
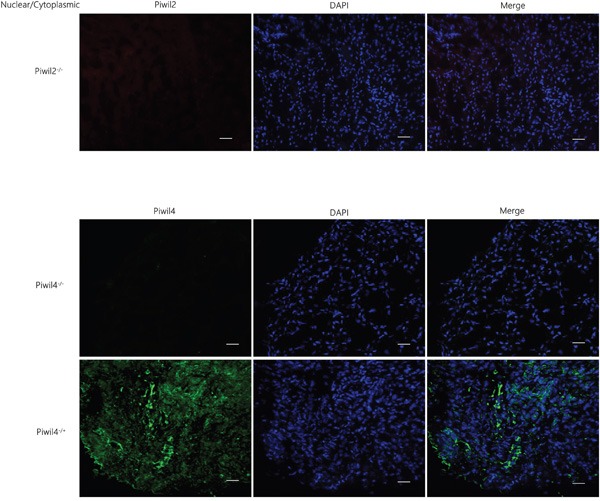
Expression of Piwil2 and Piwil4 in the normal liver tissue No expression of Piwil2 in the nuclear or cytoplasmic part of the normal liver tissue was detected. The observation of the localization and expression of Piwil4 revealed two expression patterns: cytoplasmic expression (Piwil4 n-/c+), and non-expression (Piwil4 n-/c-). Bar = 50 μm.

No expression of Piwil2 in the nuclear or cytoplasmic part of normal liver tissue was detected. The observation of the localization and expression of Piwil4 revealed two expression patterns: cytoplasmic expression (Piwil4 n-/c+), and non-expression (Piwil4 n-/c-), as shown in Figure 1.

### Correlation between the localization and expression of Piwil2 and the prognosis of HCC

In HCC tissue chips, 4 carcinoma tissue sites were missing or could not be read; 86 sites were left in carcinoma tissues. Of 90 patients, 57 died of HCC, and the median follow-up time was 14 months (1-69 months). Among the surviving 33 patients, the median follow-up time was 57 months (46-80 months).

**Figure 2:**
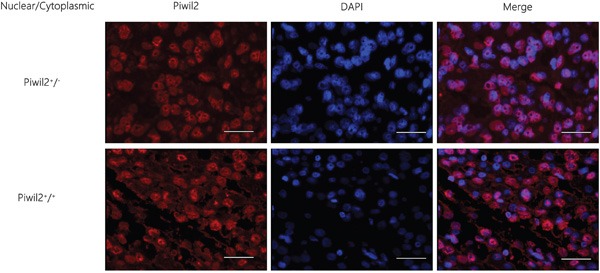
Immunofluorescence staining results of the localization and expression of a single marker Piwil2 in HCC tissues The observation of the localization and expression of Piwil2 revealed two expression patterns: nuclear expression (Piwil2 n+/c-) and nuclear and cytoplasmic expression (Piwil2 n+/c+). Bar = 50 μm.

**Figure 3:**
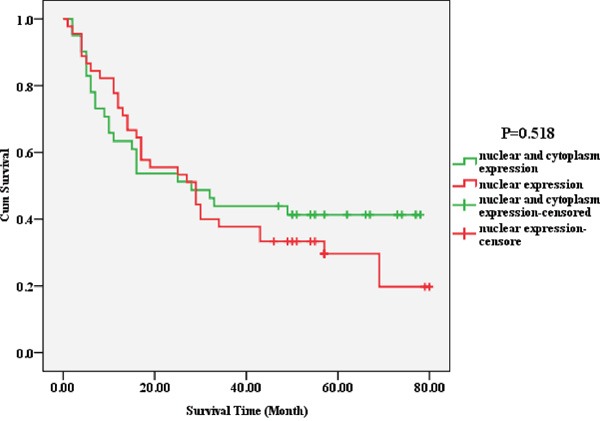
Correlation between the localization and expression of a single marker Piwil2 and the prognosis of HCC The survival rate and the overall survival of patients with the nuclear and cytoplasmic expression of Piwil2 were longer than the survival rate and the overall survival of patients with nuclear expression, but the survival time analysis showed no statistically significant difference (*P* = 0.518).

The positive expression rate of Piwil2 in the nuclear part of carcinoma tissue was 100% (86/86), significantly higher than that in the cytoplasm [47.67% (41/86)] (Fisher exact test, *P* = 0.000). The fluorescence intensity in the nucleus was higher than that in the cytoplasm. The observation of the localization and expression of Piwil2 revealed two expression patterns: nuclear expression (Piwil2 n+/c-, *n* = 45), and nuclear and cytoplasmic expression (Piwil2 n+/c+, *n* = 41), as shown in Figure 2. The survival rate and the overall survival of patients with Piwil2 nuclear and cytoplasmic expression were longer than the survival rate and the overall survival of patients with nuclear expression, but the survival time analysis showed no statistically significant difference (*P* = 0.518) (Figure 3 and Table 1 ).

**Table 1:** Correlation between the localization and expression of Piwil2 and Piwil 4 and the prognosis of HCC

Variable	Survival rate	Standard deviation	*P* value
Piwil2 localization and expression			
Piwil2 ^n+/c−^	28.9% (13/45)	4.370	0.518
Piwil2^n+/c+^	41.5% (17/41)	5.155	
Piwil4 localization and expression			
Piwil4^n+/c−^ (*n* = 20)	25.0% (5/20)	4.411	0.663
Piwil4^n+/c+^ (*n* = 51)	33.3% (17/51)	4.525	
Piwil4^n+/c−^ (*n* = 20)	25.0% (5/20)	4.411	0.242
Piwil4^n-/c+^ (*n* = 3)	66.7% (2/3)	16.602	
Piwil4^n+/c−^ (*n* = 20)	25.0% (5/20)	4.411	0.053
Piwil4^n-/c−^ (*n* = 12)	50.0% (6/12)	6.072	
Piwil4^n+/c+^ (*n* = 51)	33.3% (17/51)	4.525	0.285
Piwil4^n-/c+^ (*n* = 3)	66.7% (2/3)	16.602	
Piwil4^n+/c+^ (*n* = 51)	33.3% (17/51)	4.525	0.210
Piwil4^n-/c−^ (*n* = 12)	50.0% (6/12)	6.072	
Piwil4^n-/c+^ (*n* = 3)	66.7% (2/3)	16.602	0.592
Piwil4^n-/c−^ (*n* = 12)	50.0% (6/12)	6.072	

### Correlation between the localization and expression of Piwil4 and the prognosis of HCC

**Figure 4:**
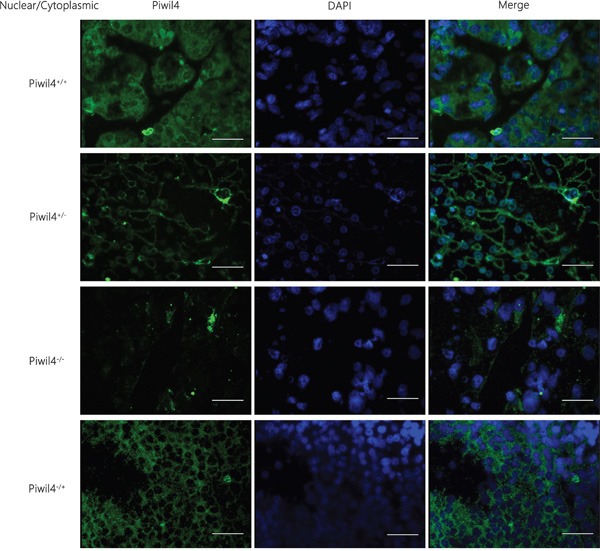
Immunofluorescence staining results of the localization and expression of a single marker Piwil4 in HCC tissues The observation of the localization and expression of Piwil4 revealed four expression patterns: nuclear expression (Piwil4 n+/c-), nuclear and cytoplasmic expression (Piwil4 n+/c+), cytoplasmic expression (Piwil4 n-/c+), and non-coexpression (Piwil4 n-/c-). Bar = 50 μm.

**Figure 5:**
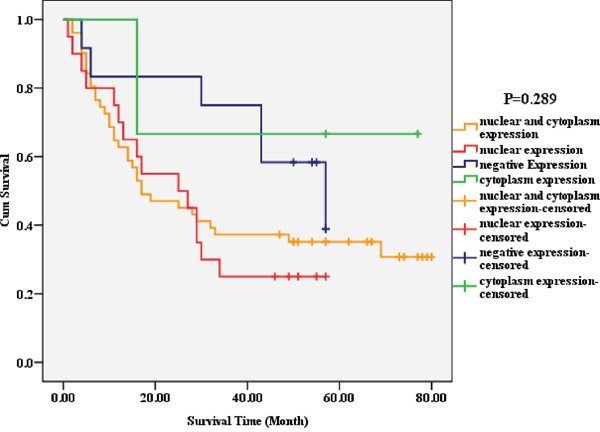
Correlation between the localization and expression of a single marker Piwil4 and the prognosis of HCC The survival rate and the overall survival for the four patterns (nuclear expression, nuclear and cytoplasmic expression, cytoplasmic expression, and non-coexpression) sequentially decreased. However, the overall survival (*P* = 0.289) and the survival curve were not statistically significantly different.

The positive expression rate of Piwil4 in the cell nucleus of carcinoma tissue was 84.52% (71/84), significantly higher than that in the cytoplasm [63.1% (53/84); chi-square test, *P* = 0.002], but the fluorescence intensity in the nucleus was weaker than that in the cytoplasm. The observation of the localization and expression of Piwil4 revealed four expression patterns: nuclear expression (Piwil4 n+/c-, *n* = 20), nuclear and cytoplasmic expression (Piwil4 n+/c+, *n* = 51), cytoplasmic expression (Piwil4 n-/c+, *n* = 3), and non-coexpression (Piwil4 n-/c-, *n* = 12) (Figure 4). The survival rate and the overall survival for the four patterns sequentially decreased. However, the overall survival (*P* = 0.289) (Figure 5) and survival curve (Table 2) were not statistically significantly different.

**Table 2:** Correlation between the localization and co-expression of molecular chaperone (Piwil2/Piwil4) and the prognosis of HCC

Variable	Survival rate	Standard deviation	*P* value
Piwil2/Piwil4 localization and expression			
Nuclear co-expression	28.9% (13/45)	4.756	0.343
Nuclear and cytoplasmic co-expression	41.5% (17/41)	5.453	
Nuclear co-expression	25.0% (5/20)	4.756	0.436
Cytoplasmic co-expression	33.3% (17/51)	21.567	
Nuclear co-expression	25.0% (5/20)	4.756	0.034
Non-coexpression	66.7% (2/3)		
Nuclear and cytoplasmic co-expression	25.0% (5/20)		0.658
Cytoplasmic co-expression	50.0% (6/12)		
Nuclear and cytoplasmic co-expression	33.3% (17/51)		0.225
Non-coexpression	66.7% (2/3)		
Cytoplasmic co-expression	33.3% (17/51)		0.904
Non-coexpression	50.0% (6/12)		

### Correlation between the localization and co-expression of molecular chaperone (Piwil2/Piwil4) and clinical pathological indicators of prognosis of HCC

**Figure 6:**
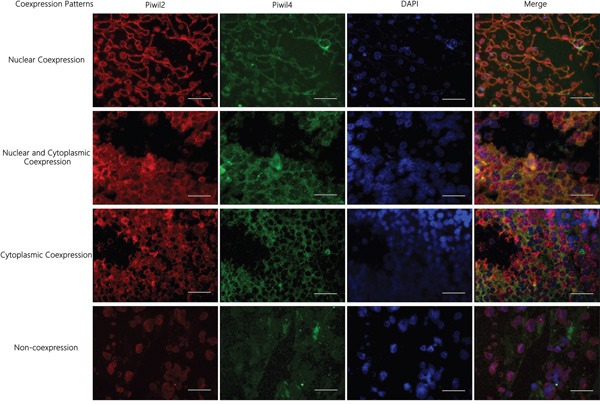
Immunofluorescence staining results of the localization and co-expression of molecular chaperone Piwil2/Piwil4 in HCC tissues Four co-expression patterns (seven co-expression ways) were observed: (1) nuclear co-expression, Piwil2^n+/c−^Piwil4^n+/c−^, Piwil2^n+/c−^Piwil4^n+/c+^; (2) nuclear and cytoplasmic co-expression, Piwil2^n+/c+^Piwil4^n+/c+^; (3) cytoplasmic co-expression, Piwil2^n+/c+^ Piwil4^n-/c+^; (4) non-coexpression, Piwil2^n+/c−^Piwil4^n-/c+^, Piwil2^n+/c+^Piwil4^n-/c−^, Piwil2^n+/c−^Piwil4^n-/c−^. bar = 50 μm.

**Figure 7:**
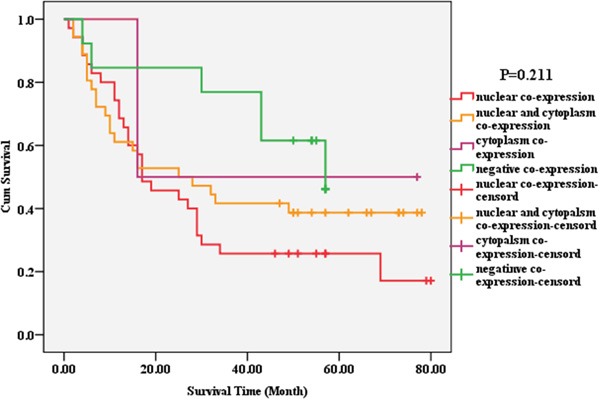
Correlation between the localization and co-expression of molecular chaperone Piwil2/Piwil4 and the prognosis of HCC The survival rates and the overall survival of the four groups (nuclear co-expression, nuclear and cytoplasmic co-expression, cytoplasmic co-expression, non-coexpression) sequentially decreased. The survival rates of patients with the four co-expression patterns were not statistically significantly different (*P* = 0.211).

Four co-expression patterns (seven co-expression ways) were observed: (1) nuclear co-expression, Piwil2^n+/c−^ Piwil4^n+/c−^, Piwil2^n+/c−^Piwil4^n+/c+^ (*n*= 35); (2) nuclear and cytoplasmic co-expression, Piwil2^n+/c+^Piwil4^n+/c+^ (*n* = 36); (3) cytoplasmic co-expression, Piwil2^n+/c+^Piwil4^n-/c+^ (*n* = 2); and (4) non-coexpression, Piwil2^n+/c−^Piwil4^n-/c+^, Piwil2^n+/c+^Piwil4^n-/c−^, Piwil2^n+/c−^Piwil4^n-/c−^ (*n* = 13) (Figure 6). The survival rates and the overall survival of the four groups sequentially decreased, which was consistent with the prognostic phenotype of patients with the localization and expression of a single marker (Piwil2 and Piwil4). They all showed a worse prognosis of positive nuclear expression. The survival rates of patients with the four co-expression patterns were not statistically significantly different (*P* = 0.211). The pairwise comparison of survival curves indicated that the survival rate and the overall survival of patients with no co-expression of Piwil2/Piwil4 were higher than those of patients with positive nuclear co-expression (53.8% vs. 22.9%). Furthermore, no statistical difference was found in the pairwise comparison of the survival curves of patients with other co-expression patterns (Table 2, Figure 7).

In the clinical pathological indicators, tumor volume (*P* = 0.029) and cTNM staging (*P* = 0.000) were related to the prognosis of HCC. The larger the volume, the higher the cTNM staging, the worse the long-term survival rate. Other indicators had no correlation with the prognosis of HCC.

## DISCUSSION

Malignant tumors severely threaten human health and obstruct social development. The metastasis, relapse, and chemoradiotherapy resistance also severely influence the patient long-term survival rate, which needs to be solved. HCC is one of the most common malignant tumors in the digestive system. The high morbidity, high fatality, and high postoperative metastasis relapse rates severely affect the long-term survival of patients with HCC [18]. Therefore, it is crucial to explore a new effective biological indicator to perform prognosis judgment or develop a new adjuvant therapy as a target.

In the evolutionary process, the phenomenon that involves the specific degradation of highly conserved and isogenous mRNA induced by exogenous or endogenous double-strand RNA (dsRNA) is called RNAi, which is an important mechanism for post-transcriptional gene silencing (PTGS) [19–21]. The PIWI family (core components of RNAi) is one of the two subfamilies (AGO and PIWI) of the Argonaute protein family, including PAZ and PIWI structural domains [3, 22]. The PIWI subfamily was first found in germ stem cells from *Drosophila* by Lin et al. in 1997 [23]. It was found to play a very important role in the self-renewal of stem cells, spermatogenesis, RNAi, transposon silencing, transcription regulation, genetic recombination, genetic programming, and so on [3–5]. The human *PIWI* gene family has four members: Piwil-like 1 (Piwil1/Hiwi), Piwil-like 2 (Piwil2/Hili), Piwil-like 3 (Piwil3), and Piwil-like 4 (Piwil4/Hiwi2) [24]. Piwil2 and Piwil4 are molecular chaperones, which cooperatively regulate the self-renewal of germ stem cells and spermatogenesis [6–8, 25].

High expression of Piwil2 protein has been reported to promote tumorigenesis, and the increased expression level of Piwil4 is also related to poor prognosis and increased risk of metastasis [10, 11, 13–16]. The study indicated that the self-renewal and proliferation regulation mechanism of germ stem cells could be involved in the occurrence of cancer stem cells (CSC) [26]. Piwil2 might be the molecular marker of pretumor stem cells [15], playing an important promoting role in tumorigenesis. CSC-like cells obtained using human fibroblasts with a single transcription factor Piwil2 (unpublished) were called Piwil2-induced cancer stem cells (Piwil2-iCSC). The Piwil2-iCSC tumor tissue was proved to be an embryonal undifferentiated carcinoma, which supported the effect of Piwil2 on the occurrence and development of tumors. The increased expression level of Piwil4 was related to soft tissue sarcoma and colon cancer metastasis. A new study also demonstrated that the lack of PIWI/PiRNA involvement would lead to the occurrence of testiculoma [27].

However, all the aforementioned studies focused only on the influence of expression of a single indicator (Piwil2 or Piwil4) on tumor biological behavior and long-term survival. They could not be used to judge the occurrence and development of tumors. No reports are available on the influence of localization and co-expression of Piwil2/Piwil4 molecular chaperone on the occurrence and development of tumors. In the present study, tissue chips combined with the immunofluorescent double staining technology were used to explore the difference in the intracellular localization and expression of Piwil2 and Piwil4. Furthermore, the difference in the prognostic evaluation between Piwil2, Piwil4, and Piwil2/Piwil4 co-expression was also recorded, and the correlation between the expression of molecular chaperone and the prognosis of HCC was analyzed.

The correlation between the localization and expression of a single marker (Piwil2 and Piwil4) and molecular chaperone and the prognosis of HCC was explored. Piwil2 and Piwil4 are not expressed in the nucleus of normal liver tissue. They are expressed only in the cytoplasm at a very low level. In the hepatocellular carcinoma tissues, Piwil2 showed two expression patterns (nuclear co-expression and nuclear and cytoplasmic expression) and Piwil4 showed four patterns (nuclear co-expression, nuclear and cytoplasmic co-expression, cytoplasmic co-expression, and non-coexpression). The survival rate and the overall survival showed a sequential decrease. The overall survival and the survival rate of patients with nuclear co-expression were lower the overall survival and the survival rate of patients with nuclear and cytoplasm co-expression, cytoplasmic co-expression, and non-coexpression, which was consistent with the result of a single marker. The survival rate and the overall survival of patients with no co-expression of Piwil2/Piwil4 were higher than those of patients with positive nuclear co-expression (53.8% vs. 22.9%) (*P* = 0.034). The nuclear co-expression of Piwil2/Piwil4 indicated worse prognosis of HCC.

Therefore, it could be deduced that the difference in the localization of Piwil2 and Piwil4 in tumor cells could be related to the malignant degree or prognostic phenotype of HCC. The expression in the cell nucleus could promote HCC to develop into a more malignant or worse prognostic phenotype. The co-expression and localization of molecular chaperone Piwil2/Piwil4 in the tumor tissue could be used as an indicator for tumor prognosis. It was conjectured that the change in the expression and localization of Piwil2/Piwil4 protein in HCC tissues could predict the prognosis. The transformation from the negative expression to the positive expression of Piwil2/Piwil4 protein in the cytoplasm indicated that the tumors were precancerous lesions or in the initial stage of tumorigenesis. The transformation from the negative expression of Piwil2/Piwil4 protein in the cytoplasm to the positive expression in the nucleus indicated that the tumor became more malignant. When the expression of Piwil2/Piwil4 protein disappeared in the cytoplasm and remained only in the nucleus, it suggested bad prognosis of HCC.

So far, the detailed molecular biological mechanism underlying the influence of Piwil2 and Piwil4 on the occurrence, development, and prognosis of HCC is still unclear, which needs further exploration. The present study was the first to perform such correlation analysis as well as explore the correlation between the cytoplasmic co-expression of Piwil2/Piwil4 and the prognosis of HCC. The molecular chaperone Piwil2/Piwil4 seems quite promising as an important molecular marker for prognosis judgment, and a single marker (Piwil2 or Piwil4) cannot be used as the indicator for the prognosis of HCC.

In the enrolled 90 cases, the patients died of HCC in the follow-up, accounting for 63.33% (57 cases) of the patients in the study. In the 33 survivors, the median follow-up time was 57 months (46-80 months), of which the follow-up time of 21 patients did not reach 5 years. The molecular biological mechanism could not be explored due to the limited sample size and follow-up time. Large-sample studies will be helpful in analyzing the correlation between the co-expression of Piwil2/Piwil4 and the prognosis of HCC. They can provide the theoretical basis for the molecular marker of early-stage diagnostic and prognostic evaluation, and targeted tumor therapies.

## MATERIALS AND METHODS

### HCC sample source

All the 90 HCC and 2 normal control samples were obtained from the tissue bank of Shanghai Outdo Biotech Co., Ltd. (HLiv-HCC180Sur-04). All the tumor samples were diagnosed with HCC and did not receive chemotherapy or radiotherapy. Two normal control samples were obtained from two male adult patients (aged 31 and 48 years) who received partial hepatectomy due to trauma. The surgery time was from August 2006 to November 2009, and the follow-up deadline was September 2013 with a period of 4-7 years. The enrolled samples included 81 males and 9 females, aged 25-73 years with a median age of 54 years. Other clinical pathological indicators and survival conditions are shown in Table 3. The clinical staging was analyzed according to the HCC international TNM staging criteria published by American Joint Committee on Cancer (2012).

**Table 3:** Clinical pathological indicators and survival conditions of 90 HCC cases

Item	*N* (%)	Item	*N* (%)
Gender		Tumor volume (cm^3^)	
Male	81 (90.00)	<125	43 (47.78)
Female	9 (10.00)	125≦V<1000	35 (38.89)
Loss	0	≧1000	11 (12.22)
Age (year)		Loss	1 (1.11)
<60	66 (73.33)	Pathological staging	
≧60	23 (25.56)	I	3 (3.33)
Loss	1 (1.11)	II	54 (60.00)
cTNM staging		III	33 (36.67)
I	11 (12.22)	Loss	0
II	29 (32.22)	Survival condition	
III	39 (43.33)	Survival	33 (36.67)
IV	3 (3.33)	Death	57 (63.33)
Loss	8 (8.89)	Loss	0

### Preparation for tissue chips

Carcinoma tissues and normal control tissues were fixed in 10% formaldehyde solution, hydrated routinely, and embedded in paraffin blocks. The embedded tissues were sliced and stained with hematoxylin and eosin, which was further diagnosed by pathologists. The target tissues were marked under a microscope. A tissue microarray (Beecher Instruments Inc.) was used to punch in the receptor blank paraffin blocks (diameter 1.5 mm). The target tissue chip was obtained from the marked location and placed into the microarray. The aforementioned steps were repeated, and finally a 92-site microarray was achieved with carcinoma and normal control tissues. The 5-μm-thick slice thus obtained was adhered onto a glass slide, making a tissue chip.

### Reagents

Goat anti-human Piwil2 polyclonal antibody (Piwil2(K-18):sc-67502) and rabbit anti-human Piwil4 polyclonal antibody (Piwil4(H-90):sc-68932) were obtained from Santa Cruz Biotechnology (Santa Cruz, USA). Cy3-labeled (red fluorescence) donkey anti-goat IgG (H+L) (A0502) was purchased from Beyotime Biotechnology (Jiangsu, China). IFKine Green-labeled (green fluorescence) donkey anti-rabbit IgG (H+L) (A24221) was obtained from Abbkine (California, USA). 4',6-Diamidino-2-phenylindole (DAPI) (D9542) was obtained from Sigma-Aldrich (Darmstadt, Germany). Anti-fluorescence quenching sealing liquid was purchased from Beyotime Biotechnology (Jiangsu, China).

### Immunofluorescence double staining

The HCC tissue chips were dried in a 60°C oven and dewaxed. After repair under high pressure by antigen, the serum was added to the block. Then, the primary antibody (Piwil2 1:100; Piwil4 1:50) was added for incubation at 4°C overnight, and the fluorescent secondary antibody was added at 37°C and incubated avoiding light for 30 min. DAPI was used to stain for 10 min, and the chips were blocked by the anti-fluorescence quenching sealing liquid. The protein precipitation was observed under a fluorescence microscope.

The tissue chips were read by two pathologists. Piwil2 protein showed red fluorescence (labeled by Cy3), and Piwil4 protein showed green fluorescence (labeled by TFKine Green). The cell nucleus showed blue fluorescence (stained by DAPI). The expression of Piwil2 or Piwil4 marker in HCC tissues was observed.

### Statistical analysis

SPSS17.0 (SPSS, IL, USA) was used to analyze the data. Kaplan-Meier survival curve and log-rank test were used to analyze the correlation between the difference in the localization and expression of a single marker (Piwil2 or Piwil4) and molecular chaperone (Piwil2/Piwil4), and the prognosis of HCC. A *P* value <0.05 was considered statistically significant.
